# Kinetic modeling of tumor regression incorporating the concept of cancer stem-like cells for patients with locally advanced lung cancer

**DOI:** 10.1186/s12976-018-0096-7

**Published:** 2018-12-27

**Authors:** Hualiang Zhong, Stephen Brown, Suneetha Devpura, X. Allen Li, Indrin J. Chetty

**Affiliations:** 1 Department of Radiation Oncology, Medical College of Wisconsin, Milwaukee, 53226 WI USA; 2 Department of Radiation Oncology, Henry Ford Health System, Detroit, 48202 MI USA

**Keywords:** Kinetic model, Tumor regression, Radiation therapy, Cancer stem-like cells, Lung cancer

## Abstract

**Background:**

Personalized medicine for patients receiving radiation therapy remains an elusive goal due, in part, to the limits in our understanding of the underlying mechanisms governing tumor response to radiation. The purpose of this study was to develop a kinetic model, in the context of locally advanced lung cancer, connecting cancer cell subpopulations with tumor volumes measured during the course of radiation treatment for understanding treatment outcome for individual patients.

**Methods:**

The kinetic model consists of three cell compartments: cancer stem-like cells (CSCs), non-stem tumor cells (TCs) and dead cells (DCs). A set of ordinary differential equations were developed to describe the time evolution of each compartment, and the analytic solution of these equations was iterated to be aligned with the day-to-day tumor volume changes during the course of radiation treatment. A least squares fitting method was used to estimate the parameters of the model that include the proportion of CSCs and their radio-sensitivities. This model was applied to five patients with stage III lung cancer, and tumor volumes were measured from 33 cone-beam computed tomography (CBCT) images for each of these patients. The analytical solution of these differential equations was compared with numerically simulated results.

**Results:**

For the five patients with late stage lung cancer, the derived proportions of CSCs are 0.3 on average, the average probability of the symmetry division is 0.057 and the average surviving fractions of CSCs is 0.967, respectively. The derived parameters are comparable to the results from literature and our experiments. The preliminary results suggest that the CSC self-renewal rate is relatively small, compared to the proportion of CSCs for locally advanced lung cancers.

**Conclusions:**

A novel mathematical model has been developed to connect the population of cancer stem-like cells with tumor volumes measured from a sequence of CBCT images. This model may help improve our understanding of tumor response to radiation therapy, and is valuable for development of new treatment regimens for patients with locally advanced lung cancer.

## Backgrounds

Radiation therapy (RT) is the treatment of choice for many lung cancer patients. The aim of an RT plan is to deliver high radiation dose to tumor volume to eliminate cancer cells without causing severe damage to surrounding healthy tissue. The efficacy of a planned treatment, often quantified by tumor control probability (TCP), depends on several patient-specific factors such as the surviving fraction of tumor cells and treatment regimens used [1, 2]. Many models and methods have been proposed to calculate the control probability [3]. In general, TCP is defined as an exponential function of the number of surviving cells after a given treatment [4]. The number of the surviving cells depends on the radiation dose and the radio-sensitivity of each cell subpopulation within the tumor. Much effort has been devoted to the development and validation of treatment response models such as, for example, the linear quadratic and the modified linear quadratic models [5, 6]. However, without calibrating radio-sensitivity parameters such as the α/β ratio for individual patients [7, 8], inter-patient differences cannot be taken into account in these models [9–11].

The radio-sensitivity of tumor cells could be heterogeneous among its subpopulations [12]. Bhaumik and Jain divided cells into the proliferating and non-proliferating compartments, and investigated the tumor growth kinetics on the basis of interactions between the two compartments [13]. Rockne et al. extended the reaction-diffusion model of tumor proliferation and invasion to a general form to include tumor response to radiation therapy [14, 15], and Ribba et al. proposed a multiscale model of cancer to investigate tumor response to radiation [16]. Zhong et al. used a pair of ordinary differential equations to capture the essential features of tumor dynamics, modeling radiation-induced kinetics in the number of tumor cells [17, 18]. Zaider showed that the amount of radiation dose necessary for obtaining a 90% TCP can be dominated by radio-resistant cells which could be as small as a millionth of the total number of tumor cells [11]. The challenge to all these approaches has been the lack of a measure of the number of these cells and their radio-sensitivities [9, 19, 20].

In the context of cancer stem-like cells (CSCs) which, by definition, are self-renewing with unlimited proliferative potential and a capacity to generate differentiated cells (i.e. cells that evolve to express proteins that confer advantages for growth, metastasis, invasion, and resistance to treatment) [21], Hillen et al. developed a mathematical model of a heterogeneous population of CSCs and non-stem tumor cells (TCs) to investigate movement between these components [19]. With this model, Yu et al. investigated the impact of different radio-sensitivities between stem-like and non-stem cancer cells, and demonstrated the importance of CSCs for tumor local control [22]. In these studies, the CSC self-renewal rate was assumed to be 1% [19, 22, 23]. These models have helped us to get a better understanding of the mechanism of heterogeneous tumor response to radiation treatment. However, the parameters used in these models are associated with large uncertainties [24]. For example, the population of CSCs has been reported to range from 0.1 to 82.5% [25–27]. It has been difficult to identify CSCs and measure their subpopulations and radio-sensitivity for individual patients. The lack of an efficient method to measure these radiobiological parameters has limited the clinical use of these models for treatment planning and monitoring response in individual patients.

With advances in radiation therapy techniques, it has become possible to measure changes in tumor volume, a major index of treatment response for solid tumors, using daily anatomical images acquired during the course of radiation treatment. In this study, we have developed a set of differential equations that includes the compartments of cancer stem-like and non-stem cells, and dead cells so that changes in each of these cell compartments affecting tumor volume during treatment can be evaluated. The analytical solution of these equations, after validating using numerical simulations, was applied to treatment regimens to form an iterative model for individual patients. Based on the tumor volumes measured from daily CBCT images, patient-specific parameters including the proportion of CSCs and their radio-sensitivities were estimated, and stabilities of these parameters were assessed. In the next sections the theoretical model is developed, and applied to a sequence of CBCT images to evaluate radiobiological parameters. The derived parameters are compared with those reported in the literature.

## Materials and methods

The observations of radiosensitivity of normal tissue stem cells by Bergonie and Tribondeau at the turn of the last century, confirmed by 100 years of experience with radiation, indicate that normal tissue stem cells are typically more sensitive to radiation than differentiated cells [28]. However, many factors such as cell quiescence, DNA repair ability, overexpression of anti-apoptotic proteins, detoxifying enzymes such as glutathione and a hypoxic niche microenvironment render CSCs to be radiation resistant and responsible for tumor growth and relapse [29–31]. CSCs shared some characteristics in common with normal tissue stem cells [32]. For example, CSCs were assumed to exhibit unlimited proliferation and a low baseline apoptosis [33]. From CSCs to differentiated cells, there are multiple stages of differentiations. Due to the limited availability of clinical and experimental data, it is impossible to model cellular interactions at all these stages. In this study we divide cells in a tumor volume observable in CBCT images into three compartments: cancer stem-like cells, non-stem tumor cells (TC) and dead cells (DC).

### A kinetic model of tumor response to radiation treatment

To model the transient status of hierarchical cancer cells, each cell subpopulation is described as a compartment, and different compartments are connected by transitional events with fixed rates. For example, a cancer stem-like cell is assumed to divide at a constant rate, to either two CSCs (symmetric division) or one CSC and one TC (asymmetric division). Let u(t) and v(t) denote the numbers of CSCs and TCs per unit volume, and w(t) the number of DCs before being moved out of the tumor volume. Let m_u_ and m_v_ denote the division rates of CSCs and TCs. Let δ represent the probability of the symmetry division which is equivalent to the rate of the CSC self-renewal, and let *ϒ*_*u*_ and *ϒ*_*v*_ denote the rates of the radiation-induced cell death for CSCs and TCs, m_a_ the rate of the programmed cell death for TCs without irradiation, and *ϒ*_*w*_ the clearance rate of DCs. The interaction of the three compartments (CSC, TC and DC) can be mathematically represented by

$$ {\dot{u}}_t=\delta {m}_uu(t)-{\varUpsilon}_uu(t) $$

1 $$ {\dot{v}}_t=\left(1-\delta \right){m}_uu(t)+{m}_vv(t)-\left({m}_a+{\Upsilon}_v\right)v(t) $$

$$ {\dot{w}}_t={\varUpsilon}_uu(t)+\left({m}_a+{\varUpsilon}_v\right)v(t)-{\varUpsilon}_ww(t) $$

To apply this set of differential equations to daily treatment regimens which specifies when the treatment will be delivered and how much radiation dose will be used during the course of radiation therapy, these equations have to be solved explicitly. Let SF_u_ and SF_v_ denote the surviving fraction of cells in u(t) and v(t) after being irradiated by one daily fraction, 2 Gy. *ϒ*_*u*_ and *ϒ*_*v*_ can be represented as *ϒ*_*u*_ =  − ln *SF*_*u*_ and *ϒ*_*v*_ =  − ln *SF*_*v*_. The parameters *m*_*u*_, *m*_*v*_ are related to the tumor doubling times T_u_ and T_v_ by *m*_*u*_ =  *ln* 2/*T*_*u*_ and *m*_*v*_ =  *ln* 2/*T*_*v*_, and the clearance rate of DCs is *ϒ*_*w*_ =  *ln* 2/*T*_*h*_, and *m*_*a*_ =  *ln* 2/*T*_*a*_ where T_h_ is the half time for the dead cell recycling and T_a_ is the half time for the programmed cell death. Following the mathematical reasoning illustrated in [17], the differential equations can be explicitly solved as

$$ u(t)=u(0){e}^{At} $$

2 $$ v(t)=\frac{Bu(0)}{A-C}{e}^{At}+\left(v(0)-\frac{Bu(0)}{A-C}\right){e}^{Ct} $$

$$ w(t)=\left(\frac{\varUpsilon_uu(0)}{A+{\varUpsilon}_w}+\frac{Bu(0)}{A-C}\frac{m_v+{\varUpsilon}_v}{A+{\varUpsilon}_w}\right)\left({e}^{At}-{e}^{-{\varUpsilon}_wt}\right)+\frac{m_v+{\varUpsilon}_v}{C+{\varUpsilon}_w}\left(v(0)-\frac{Bu(0)}{A-C}\right)\left({e}^{ct}-{e}^{-{\varUpsilon}_wt}\right)+w(0){e}^{-{\varUpsilon}_wt} $$

where $A=\frac{\delta ln2}{T_u}+\ln {SF}_u$, *B* = (1 − *δ*) *ln* 2/*T*_*u*_, and$C=\mathit{\ln}2\left(\frac{1}{T_v}+\frac{1}{T_a}\right)+\ln {SF}_v$. Let *t* = 1, then the resultant solution can be applied to each RT fraction or non-RT days: for non-RT days SF_u_ and SF_v_ will be set to 1; otherwise, these parameters will be optimized. The resultant kinetic model is executed on each day, and the computed numbers of the cells in each compartment at day i + 1 can be written as:

$$ {U}_{i+1}={U}_i{e}^A, $$

3 $$ {V}_{i+1}={U}_i\frac{B}{A-C}{e}^A+\left({V}_i-\frac{B}{A-C}{U}_i\right){e}^C $$

$$ {W}_{i+1}={U}_i\left(\frac{-\ln {SF}_u}{A+\mathit{\ln}2/{T}_h}+\frac{B}{A-C}\frac{\mathit{\ln}2/{T}_v-\ln {SF}_v}{A+\mathit{\ln}2/{T}_h}\right)\left({e}^A-{e}^{-\mathit{\ln}2/{T}_h}\right)+\frac{\mathit{\ln}2/{T}_v-\ln {SF}_v}{C+\mathit{\ln}2/{T}_h}\left({V}_i-\frac{B}{A-C}{U}_i\right)\left({e}^c-{e}^{-\mathit{\ln}2/{T}_h}\right)+{W}_i{e}^{-\mathit{\ln}2/{T}_h}. $$

Since CBCT imaging was used in patient treatment, radiation impact on cell survival can be assessed on a daily basis. By iterating Eq. (3) for each day, the number of the survived cells in each compartment can be calculated, and consequently the number of the total survived cells can be compared with the CBCT-measured results.

### The numerical solution of the kinetic model

With any given parameter, Eq. (1) can be numerically solved. The numerical solution can be used to validate those generated by the iterative functions in Eq. (3). Specifically, an Euler method was applied to Eq. (1) with the procedure sketched below:

Let X(t) = (u(t), v(t), w(t)), and let ${\dot{X}}_t=f\left(t,X\right)$ with *f*(*t*, *X*) defined by the differential Eq. (1). Let the initial condition satisfy X(0) = X_0_ = (u_0_, v_0_, 0). For k = 0, . . ., n-1, let a = k, b = k + 1, and h = (b – a)/m. Then for *i* = 0, …, m,

4 $$ {X}_{i+1}={X}_i+ hf\left({t}_i,{X}_i\right);{t}_i=a+ ih $$

The vector X_m_ will be an approximation to the solution X(k + 1) of the differential equations, for each k = 0,. .., n-1. By adding all the components in X_m_ together, the number of the total tumor cells can be calculated and compared to the results derived from the analytical iterative model in Eq. (3).

Equation (4) can be used to validate the analytical solution (3), but is not suitable to be used for parameter optimization. Note that the numerical method costs more computation time than using the explicit, analytic solution, and also the limited precision of the numerical solution may compound the uncertainties of the parameter derivation. After verification of the consistency of the two models, only the analytical model in (3) was used to derive radio-biological parameters. The simulation of treatment regimens and its related parameter optimization were programmed using C++ on a Linux workstation.

### Tumor volume measurement from CBCT images for lung cancer patients

With the approval from the Institutional Review Board (IRB) of Henry Ford Health System (HFHS), five lung cancer patients treated at HFHS were investigated in this study. Among the five patients, 2 are with adenocarcinoma and 3 with squamous cell cancer (see Table 1). All of them were at stage III, and treated 33 fractions, 2 Gy/fraction using 6 MV IMRT. Daily CBCT images were acquired to help setup patients and monitor tumor regression. Gross tumor volumes (GTVs) were contoured by physicians on each of these CBCT images, and the contoured volumes were calculated using the Eclipse planning system (Varian Medical Systems, Palo Alto, CA). The dates for RT and CBCT imaging, and the tumor volume measured on each of these images were written into an input file for the kinetic model. Most of the patients have their treatment course completed within 47 days, except patient A2 who had no treatment for one week, resulting in the RT course extended to 55 days.

**Table 1:** Characteristics of the five patients investigated in this study, where Adeno Ca represents adenocarcinoma, LUL, RUL and RLL represent left upper lobe, right upper lobe, and right lower lobe, respectively, and GTV represents gross tumor volume

Patient	Age	Sex	Stage	Histology	GTV (cm^3^)	Location	Treatment
A1	77	Male	IIIA	Adeno Ca	162.5	LUL	RT/chemo
A2	80	Male	IIIB	Adeno Ca	177.1	RUL	RT/chemo
S1	65	Female	IIIA	Squamous	115.9	RLL	RT/chemo
S2	78	Male	IIIB	Squamous	77.6	RUL	RT/chemo
S3	80	Female	IIIA	Squamous	91.8	RUL	RT/chemo

### A least squares fitting method to optimize the model

For the five patients investigated in this study, the tumor volumes delineated in 33 CBCT images were normalized to that in the first CBCT to obtain the rate of tumor regression for each of these RT days. The coefficients δ, p, T_h_, and SF_u_ in the model were estimated by comparing the modeled tumor volumes with those measured from CBCT images using a least squares fitting method. With the assumption that tumor volume is proportional to the number of its clonogens with a linear constant coefficient [34], an objective function can be defined by

5 $$ R=\sqrt{\frac{1}{n}{\sum}_{i=1,\dots, n}\left|\frac{M_i}{M_0}-\frac{G_i}{G_0}\right|} $$

where M_i_ is the tumor volume measured from the *i-th* CBCT scan, and *G*_*i*_ = *U*_*i*_ + *V*_*i*_ + *W*_*i*_ is its corresponding cell number predicated by the kinetic model (3). At the beginning of treatment, it is assumed that W_0_ = 0 and the proportion of CSCs is denoted by *p* = *U*_0_/(*U*_0_ + *V*_0_). Let T_u_, T_v_, and T_a_ share the same doubling time as suggested in [22–24], and the surviving fraction SF_T_ of the total cells be written as

6 $$ {SF}_T={pSF}_u+\left(1-p\right){SF}_v. $$

The two terms in the right-hand side of Eq. ((6)) represent the survival fractions of stem and non-stem cells, respectively. With the parameters T_u_ and SF_T_ measured for different types of cancer cells, the other parameters were derived by minimizing the function R in Eq. ((5)) using the least squares fitting method.

It has been reported that the cell surviving fraction under 2 Gy is 0.90 and the cell division time T_div_ is 60 h for squamous cell cancer [35, 36]. For adenocarcinoma (A549), it has been reported that the surviving fraction is 0.62 and cell division time is 22 h [37, 38]. However, T_div_ is not appropriate to be used directly as the tumor doubling time in Eq. (3) due to the existence of quiescent cells. On the other hand, the time (T_vol_) required to double tumor volume was reported to be 166.3 days on average over 237 patients, with 221.6 days for adenocarcinoma, 115.2 days for squamous cell carcinoma [39]. Note that the measured time (T_vol_) is based on the resultant volumes that already included the factor of cell loss, and therefore, does not represent the cell division time required in Eq. (3).

Different from the above two rates of tumor growth, the potential doubling time (T_pot_), calculated as the ratio of the time for DNA synthesis and the proportion of cells synthesizing DNA, takes into account the number of quiescent cells, and is a more objective index estimating proliferation occurring during a course of radiation treatment [40]. Typical values for T_pot_ have been found to be 6.2 days for squamous and 7.1 days for adenocarcinoma cells [22, 41]. In the next section, we will compare the potential doubling time with the cell division and volume doubling times, and evaluate the impact of tumor growth uncertainties on the optimized parameters. We will also measure the cell survival fraction and compare the measured results with those reported in literature for squamous cell carcinoma.

## Results

### Results of the kinetic modeling

Based on the potential doubling times reported for adenocarcinoma and squamous cell cancer, the least squares fitting method was applied to the CBCT image sequences acquired from the five patients to minimize the objective function R. The resultant residues were found to be less than 8.2%. The optimized parameters were listed in Table 2. For the patients with squamous cell cancer, the derived proportions of CSCs are 0.229 on average, the probability of the symmetry division are 0.036 and the surviving fractions of CSCs are 0.981, respectively. For the adenocarcinoma patients, the average probability of the symmetry division is 0.09, and the average surviving fractions and proportions of CSCs are 0.945 and 0.408, respectively.

**Fig. 1:**
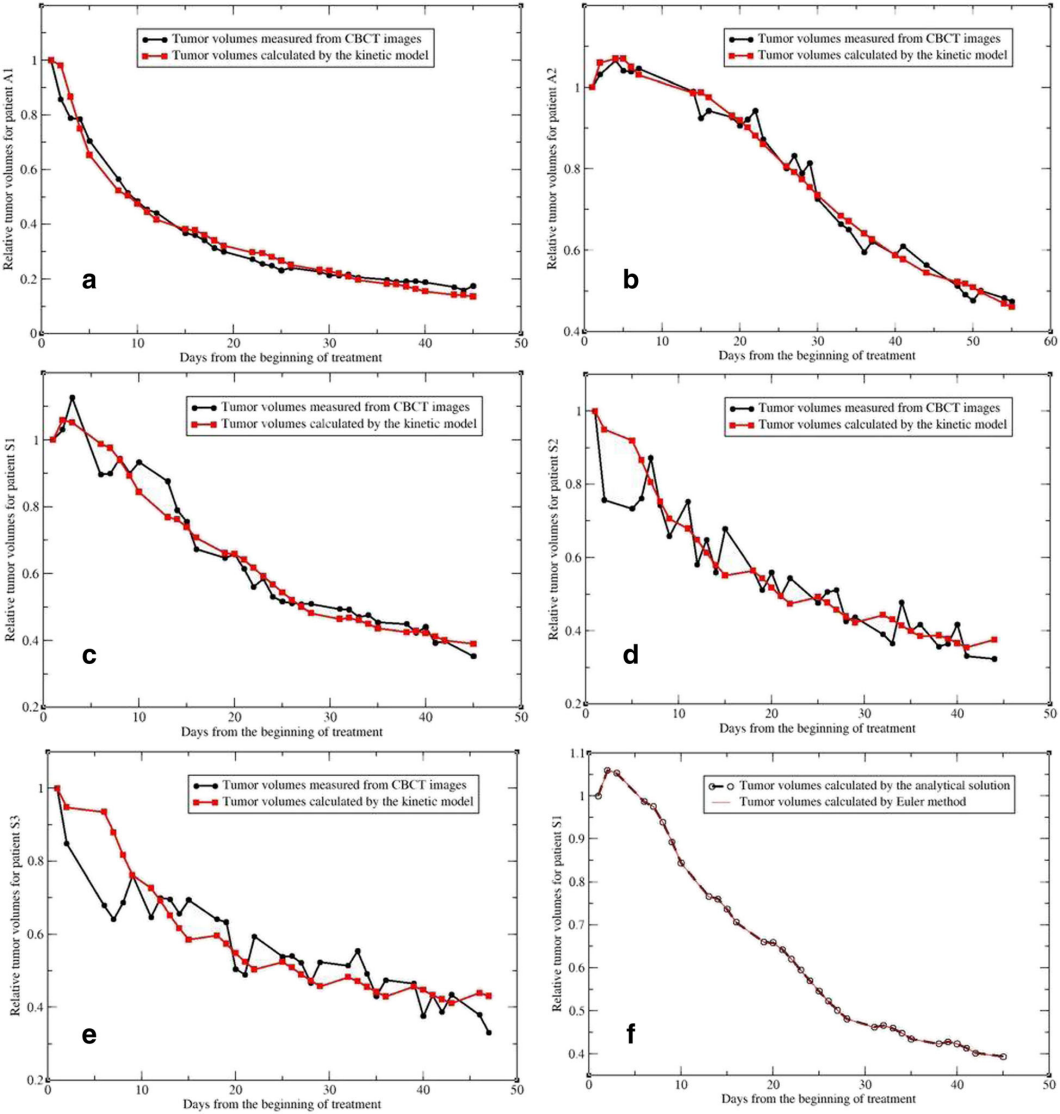
(**a**-**e**) The tumor volumes calculated by the kinetic model compared with those measured from CBCT images for the five patients; (**f**) The tumor volumes calculated with the analytical and numerical solutions (3) and (4), respectively. Note that the x-axis represents the treatment dates, and the y-axis highlights the range of tumor regression

With these optimized parameters, the number of tumor cells in each compartment was calculated using the iterative function in Eq. (3). The total number of the tumor cells computed for each day was normalized to that on the first RT day to get compared with the relative tumor volumes measured from 33 CBCT images. The model-calculated and measured tumor volumes, relative to their values on the first RT day, were shown in Fig. 1. The fitting errors in percentage are 2.6 ± 2.33, 2.1 ± 1.75, 3.0 ± 2.81, 4.95 ± 4.72, 5.73 ± 4.78 for the five patients, respectively.

With the same optimized parameters, the number of the tumor cells on each of the RT days was calculated using Eqs. (3) and (4), respectively. The computed results were plotted as shown in Fig. 1f. Note that while the Eqs. (3) and (4) were derived with two totally different methods, the numbers of the tumor cells on each day computed by the two equations are almost identical. This has verified that both the analytical and numerical solutions have been implemented correctly in this study.

### Impact of tumor doubling time on the derived parameters

**Fig. 2:**
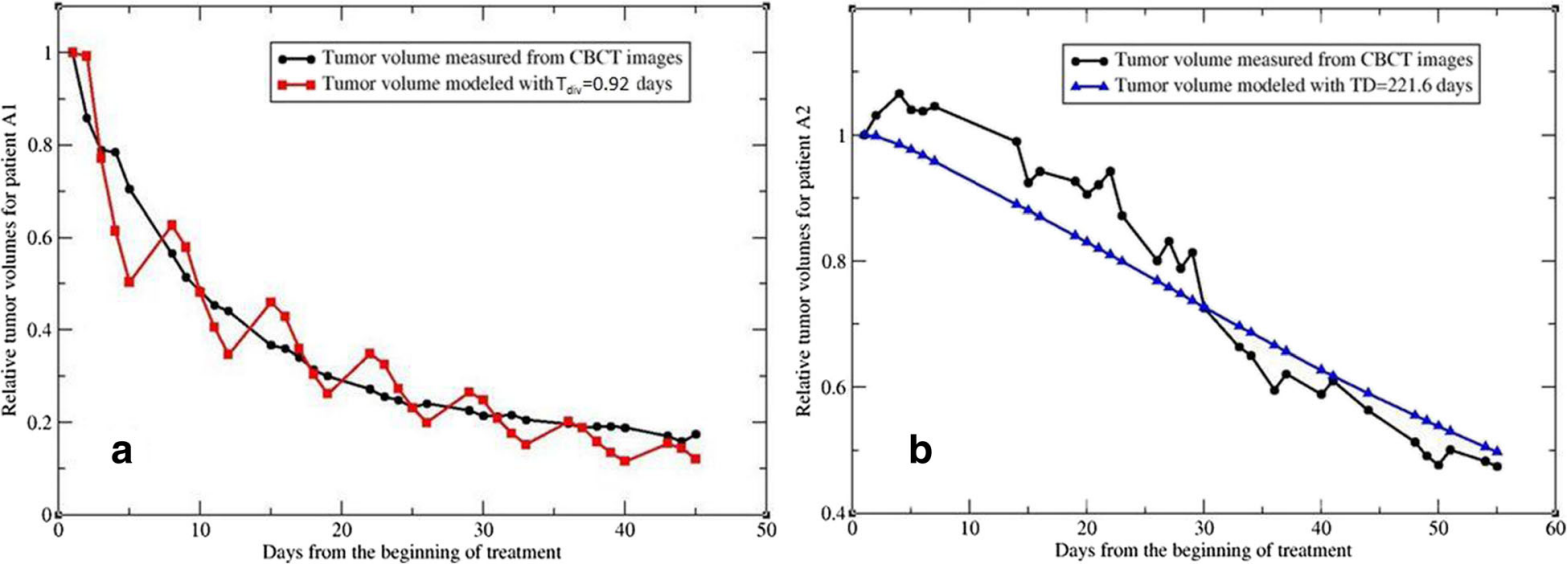
The measured tumor volumes compared with the model-calculated volume using (**a**) the cell division time (T_div_ = 0.92) for patient A1, and (**b**) the volume doubling time (T_vol_ = 221.6) for patient A2

In the previous section, the T_pot_ values reported in literature were assigned to the parameter T_u_. When these values were replaced by the cell division time T_div_, which is 22 h for adenocarcinoma, the tumor volumes calculated by the kinetic model would be in a zigzag shape as shown in Fig. 2a (in red). The rapid increase after each weekend is due to the short doubling time used in the model, no matter how the other parameters were optimized. This example demonstrated that without using an appropriate tumor volume doubling time, the kinetic model cannot fit to the measured tumor volumes accurately, and T_div_ is not suitable to be used as T_u_ in this model. In contrast, the tumor volume doubling time (T_vol_ = 221.6 days) is much longer than the potential doubling time [39]. Use of the volume doubling time makes the tumor growth over weekend negligible, and the optimized curves are very smooth. However, the calculated volumes still could not fit to the measured tumor volumes (Fig. 2b). Compared to T_div_ and T_vol_, the measured T_pot_ values showed the best fit for the kinetic model (Fig. 1).

The fitness of different doubling times used in the model was quantitatively assessed for the five patients. It has been found that the T_pot_-based residual values R are less than or comparable to those generated by the T_div_ or T_vol_-based model, for each of these patients. All the optimized residual values were shown in Table 3.

### Stability of the kinetic model at the optimized parameters

**Fig. 3:**
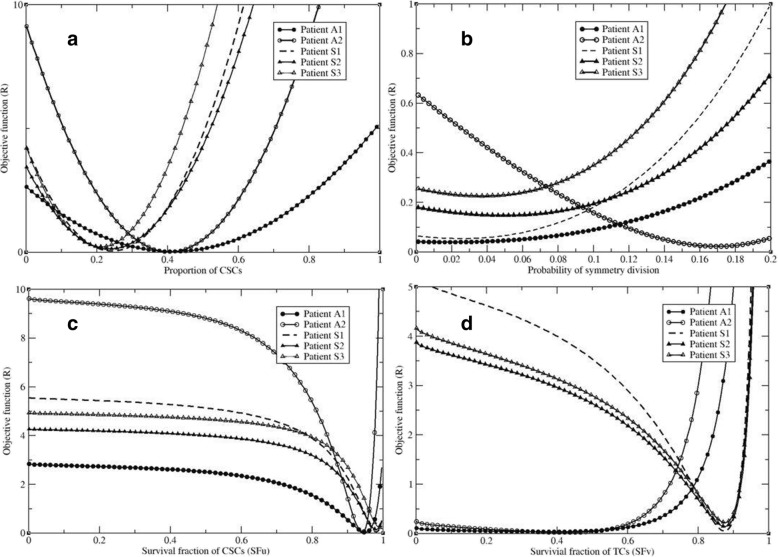
The stability of the optimized parameters: (**a**) the proportion of CSCs, (**b**) probability of the symmetry division, (**c**) surviving fraction of CSCs, and (**d**) surviving fraction of TCs

With the proportion of the CSCs (p) changed from 10^− 3^ to 1.0, and the other parameters fixed at the optimized values as shown in Table 2, the resultant function R was plotted in Fig. 3a. It can be found that only one minimum point exists for each patient. Similarly, let the probability of the symmetry division (δ) change within a reasonable range of 0.001 to 0.2 as reported in [22, 23], and the surviving fraction of CSCs change within the range of 0.001 and 1.0, the objective function R was computed with results illustrated in Fig. 3b and c, respectively.

**Table 2:** Parameters derived from the CBCT-measured tumor volumes for the five patients

Patient	δ	p	SF_u_	SF_v_	T_h_ (day)	R
A1	0.018	0.416	0.950	0.385	1.5	0.035
A2	0.162	0.400	0.942	0.406	8.1	0.027
S1	0.024	0.242	0.980	0.874	1.1	0.041
S2	0.048	0.235	0.978	0.876	0.5	0.067
S3	0.037	0.211	0.986	0.877	0.5	0.082

**Table 3:** The residues of the optimized objective function R with the potential doubling time (T_pot_), cell division time (T_div_) and volume doubling time (T_vol_) used, respectively, for the five patients

R	A1	A2	S1	S2	S3
T_u_ = T_pot_	0.035	0.027	0.041	0.067	0.082
T_u_ = T_div_	0.192	0.933	0.074	0.173	0.269
T_u_ = T_vol_	0.031	0.292	0.051	0.156	0.185

Figure 3 showed that the kinetic model is stable at the optimized points for the parameters p and SF_u_, where the minimum points for the function R were clearly demonstrated. In contrast, the rate of the CSC self-renewal was shown to be sensitive to the objective function R.

### Verification of the cell surviving fraction

Tumor cell radiosensitivity is a major determinant of tumor response to radiation treatment [2]. The following experiment was conducted to verify the surviving fraction of cancer cells under radiation. The cell lines of NCI-H2170, originated from a lung patient with squamous cell carcinoma, were cultured and investigated. The first step of the experiment was to evaluate the growth of this type of cells without irradiation. The cell lines were counted on 5 different days, 3 samples per day with 1.6 million cells used at the beginning of the experiments (see Table 4). The second step is similar to step 1 except that the cells were irradiated by 2 Gy after attached to the plate. The numbers of the cells were counted 3 times (days 2, 6, and 9), 3 samples per day. The ratios of the cell numbers in step 2, relative to those in step 1 are 0.78, 0.56 and 0.44 for the three days.

**Table 4:** N_1_ and N_2_ representing the average numbers of the sample cells counted in step 1 (non-irradiated) and step 2 (irradiated by 2 Gy), respectively. X indicates no experimental data for that day

	Day 2	Day 3	Day 4	Day 6	Day 9
N_1_ (million)	2.78	3.24	4.32	8.27	21.2
N_2_ (million)	2.16	X	X	4.62	9.38
N_2_/N_1_	0.78	X	X	0.56	0.44

**Fig. 4:**
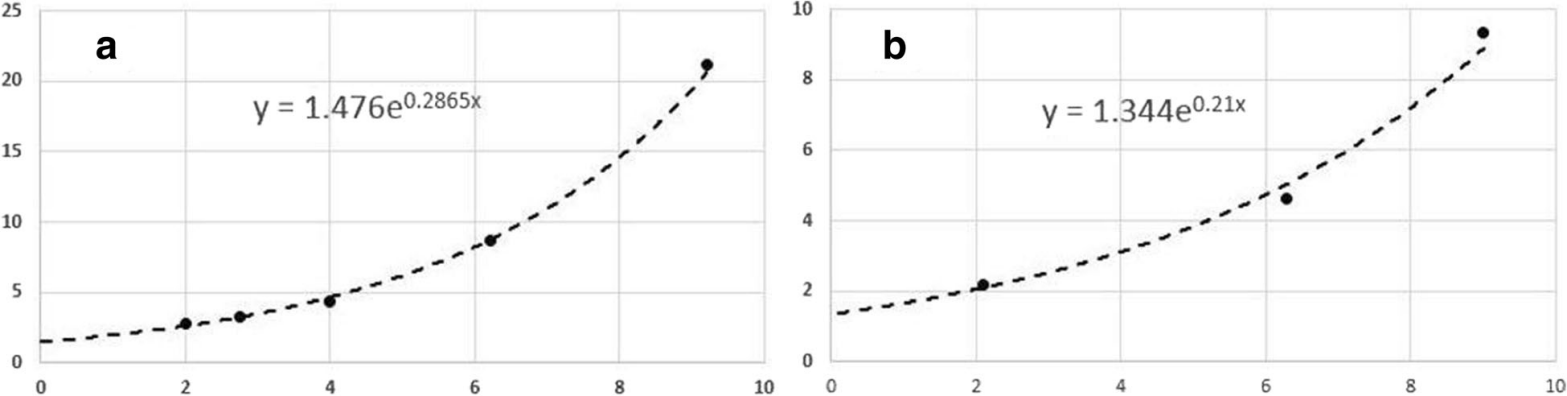
(**a**) The number of cells without irradiation and (**b**) the number of survived cells after irradiated by 2 Gy were counted and fitted with the exponential function. Note that the x-axis represents the dates for cell counting, and the y-axis represents the number of the survived cells in millions

Due to the effect of cell repopulation, the ratio N_2_/N_1_ continuously decreases. Exponential extrapolations showed that the number of the survived cells on day 0 is 1.476 (million) for the non-irradiated experiments, and 1.344 (million) for the irradiated experiments. Consequently, the surviving fraction after 2 Gy, SF_2_, counted as the ratio N_2_/N_1_ on day 0, is 0.91. The fitted exponential formula in Fig. 4a showed that the cell division time is ln2/0.2865 = 2.42 days or 58.1 h. The measured values (0.91 and 58.1 h) are consistent to 0.90 for SF_2_ and 60 h for the cell division time reported for squamous cell carcinoma [35, 36].

## Discussion

Radiation dose for locally advanced lung cancer patients is often limited by radiation toxicity to healthy organs due to the large size of tumor volume, and the 5-year surviving rate for these patients is only about 5~ 14%. For non-small cell lung cancer (NSCLC) patients, tumor volume usually will shrink after a few treatment fractions. Consequently, the original treatment plan could be reassessed to determine if the remaining tumor can be better targeted[42, 43]. The goal, of course, is to use the minimal dose that is necessary to eliminating cancer cells in the remaining tumor target. Cancer stem-like cells complicate treatment planning because of their reported radiation resistance [44]. Kinetic modeling of CSCs may provide a better understanding of tumor response to radiation therapy, and is valuable for developing new treatment regimens for cancer treatment [26].

In the context of CSCs, the minimal dose required may depend on the proportion of CSCs and their radio-sensitivities. The fraction of the stem cells is central to the cancer stem cell debate [26]. Although often a small population has been reported to be present, e.g. 0.2~0.8% for pancreatic cancer [45] and ~ 1% for prostate cancer [46], a much larger fraction (27%) with tumor initiation capability may be present for other cancers, such as melanoma [47]. Indeed, in some tumors almost the entire tumor could be comprised of CSCs. Other factors also could affect the fraction of CSCs in a tumor. For example, CSC numbers could depend on a tumor’s stage [19] and the frequency of CSCs might increase during tumor progression [27]. In the current study, the proportion of CSCs derived from the analyses was as high as 40% for the locally advanced NSCLC patients. It was found that the average proportions of stem-like cells were 22.9 and 40.8% for the patients with squamous cell cancer and with adenocarcinoma, respectively. Furthermore, in previous studies it was presumed that the CSC self-renewal rate δ is 0.01 [19, 23]. In this study we found that the rate is between 0.018~0.048 for 4 out of the five patients except patient A2 who has a relatively large δ value (0.16). This patient missed treatment for one week after RT started, but the reason for the relatively large δ needs to be further investigated.

While many kinetic models of cancer stem cells have been developed to investigate cell biological dynamics [23, 24], quantifying the population of the stem cells, and measuring parameters used in these models have proved to be difficult [21, 48]. We extended the kinetic models to include a compartment of dead cells so that changes in tumor volume observed from a sequence of CBCT images can be incorporated into the investigation of radio-biological parameters. This requires the mathematical model to be relatively simple, and in this study, components such as transitional progenitors were not explicitly modeled. For the same reason, many other factors such as cell quiescence, intercellular signaling, immune function and tumor microenvironment were not specifically modeled in this study [49–51]. Nevertheless, the set of ordinary differential equations modeled three representative compartments, and characterized the time evolution of each cell subpopulation with the analytic solution aligned with the day-to-day tumor volume changes. As a preliminary study, this work demonstrated the potential to correlate the properties of CSCs with tumor volume changes measured from anatomical images acquired during the course of radiation treatment.

It should be mentioned that due to the quality of CBCT images, uncertainties may exist in the contoured tumor volumes, especially for patients with tumor attached to chest walls or mediastinal structures. However, these uncertainties averaged over 33 fractions may have limited impact on the residual function R. Furthermore, with more anatomical and functional images such as magnetic resonance imaging (MRI) and PET/CT being used during the course of radiation therapy, the tumor volumes could be measured more precisely and consequently will help improve the accuracy of the kinetic model. On the other hand, new therapies that target CSCs are under development. Antibody-based constructs that target cell surface proteins expressed on CSCs, natural products like resveratrol and curcumin, some small molecule inhibitors, and classical drugs such as metformin have all shown potential cytotoxicity against CSCs [52]. Since radiation therapy is a standard treatment for many lung cancers, these agents will be adjuvant to RT and since CBCT images are often acquired during the course of fractionated RT, tumor volume data used for the current analysis could be available for subsequent evaluation of the efficacy of new therapies that target CSCs. To our knowledge, this is the first kinetic model that relates the changes in proportion of cancer stem-like cells to tumor volume changes measured in lung cancer patients. This model could also be applied to patients with other cancers, treated with therapies other than radiation, or with measured tumor volumes using other imaging modalities.

## Conclusion

A mathematical model has been developed to relate the kinetics of cancer stem-like cell proportions to tumor volume changes observed from a sequence of CBCT images acquired during the course of radiation treatment. The model integrated with clinical data may help in our understanding of the underlying mechanism of tumor response to radiation treatment and perhaps other therapies that target CSCs, and therefore is valuable in the development of new treatment regimens for individual patients.

## Abbreviations

CBCT: Cone-beam computed tomography
CSC: Cancer stem-like cell
DC: Dead cell
IMRT: Intensity-modulated radiation therapy
MRI: Magnetic resonance imaging
NSCLC: Non-small cell lung cancer
PET: Positron emission tomography
RT: Radiation therapy
TC: Non-stem tumor cell
TCP: Tumor control probability
